# An Intelligent System Approach for Asthma Prediction in Symptomatic Preschool Children

**DOI:** 10.1155/2013/240182

**Published:** 2013-03-14

**Authors:** E. Chatzimichail, E. Paraskakis, M. Sitzimi, A. Rigas

**Affiliations:** ^1^Department of Electrical and Computer Engineering, Democritus University of Thrace, 67100 Xanthi, Greece; ^2^Department of Pediatrics, Democritus University of Thrace, 68100 Alexandroupolis, Greece

## Abstract

*Objectives*. In this study a new method for asthma outcome prediction, which is based on Principal Component Analysis and Least Square Support Vector Machine Classifier, is presented. Most of the asthma cases appear during the first years of life. Thus, the early identification of young children being at high risk of developing persistent symptoms of the disease throughout childhood is an important public health priority. *Methods*. The proposed intelligent system consists of three stages. At the first stage, Principal Component Analysis is used for feature extraction and dimension reduction. At the second stage, the pattern classification is achieved by using Least Square Support Vector Machine Classifier. Finally, at the third stage the performance evaluation of the system is estimated by using classification accuracy and 10-fold cross-validation. *Results*. The proposed prediction system can be used in asthma outcome prediction with 95.54 % success as shown in the experimental results. *Conclusions*. This study indicates that the proposed system is a potentially useful decision support tool for predicting asthma outcome and that some risk factors enhance its predictive ability.

## 1. Introduction

Asthma is a chronic inflammatory disorder of the airways characterized by an obstruction of airflow, which may be completely or partially reversed with or without specific therapy [1]. Airway inflammation is the result of interactions between various cells, cellular elements, and cytokines. In susceptible individuals, airway inflammation may cause recurrent or persistent bronchospasm, with symptoms like wheezing, breathlessness, chest tightness, and cough, particularly at night or after exercise. Asthma is a disease with polymorphic phenotype affected by several environmental and genetic factors which both play a key role in the development and persistence of the disease [2, 3]. Among these factors family history of asthma, presence of atopic dermatitis or allergic rhinitis, wheezing episodes during childhood, maternal smoking during pregnancy, and several prenatal and environmental factors are included [4–7].

Most children who suffer from asthma develop their first symptoms before the 5th year of age [8]. However, it is difficult to discriminate asthma from other wheezing disorders of the childhood because the symptoms are similar. Thus, children with asthma may often be misdiagnosed as having a common cold, bronchiolitis, or pneumonia. For the diagnosis of asthma a detailed medical history and physical examination along with a lung function test is usually required. On the other hand, lung function test is hard to be performed in children younger than five years old. 

In preventive medicine, the value of a test lies in its ability to identify those individuals who are at high risk of an illness and who therefore require intervention while excluding those who do not require such intervention. The accuracy of the risk classification is of particular relevance in the case of asthma disease. Early identification of patients at high risk for asthma disease progression may lead to better treatment opportunities and hopefully better disease outcomes in adulthood [9–13]. 

Several efforts have been made by different groups to discover a safe way of prediction of asthma outcome such as asthma index API or modified asthma index mAPI in children younger than five years old [14, 15]. To the knowledge of the authors, this is the first study where machine learning techniques are used in the prediction of persistent asthma. However, Principal Component Analysis (PCA) has been used in several medical studies as for instance to evaluate the multivariate association between functional microvascular variables and clinical-laboratorial-anthropometrical measurements [16]. Moreover, in the study of [17], multivariate projection techniques have been utilized to reveal how inflammatory mediators demonstrate a distinct pattern of response to traumatic brain injury in humans. Finally, in [18], PCA was used for Gait Kinematics Data in Acute and Chronic Stroke Patients. Least Square Support Vector Machine (LSSVM) classifiers have been used with success for diagnosis of lung cancer [19] and in a hepatitis diagnosis system [20].

PCA provides a powerful method for exploring complex datasets with multiple variables and missing data points with relatively small numbers of observations [21]. LSSVM is a robust and reliable classifier system and has the ability to perform fast classification. For these reasons, those two techniques have been chosen for this study [22]. 

In this paper an intelligent system approach for asthma prediction outcome is presented. The system consists of three stages: (a) feature extraction and reduction through PCA, (b) pattern classification by using LSSVM classifier, and (c) the performance evaluation of the classifier by means of accuracy, sensitivity, specificity, and 10-fold cross validation. The paper is organized as follows.  Section 2.1 presents the experimental dataset which has been used for this study. In Section 2.2 brief description of the PCA is shown, while in Section 2.3 the LSSVM classifier is introduced. In Section 2.4 the proposed prediction system is presented, while the results are shown in Section 3. The discussion and the final conclusions are described in Sections 4 and 5, respectively.

## 2. Methods

### 2.1. Clinical Data

Data from 148 patients from the Pediatric Department of the University Hospital of Alexandroupolis, Greece were collected during the period 2008–2010 and recorded. A group of 148 patients who received a diagnosis of asthma were studied prospectively from the 7th to 14th year of age. All patients with missing data were excluded from the present study, leaving a total of 112 patients.

A case history, including data on asthma, allergic diseases, and lifestyle factors was obtained by questionnaires. The participants (parents and their children) answered questions regarding asthmatic and allergic symptoms, wheezing episodes until the 5th year, pet keeping, family members, parental history, and some other useful information. The prognostic factors that were used in the questionnaire have been derived from previous studies [2–10]. A total of 46 prognostic factors have been considered and they are summarized in Table 1. For some of them a kind of encoding was required in order to be efficiently utilized for the current investigation. Their encoding is presented in Table 2.

### 2.2. Principal Component Analysis for Feature Reduction

In the present study, the dimension of the input vector is large, while at the same time the components of the vectors are strongly correlated. It is, therefore, useful in this case to reduce the dimension of the input vectors. An effective procedure to perform this operation is to employ the PCA method. This technique has three effects: it orthogonalizes the components of the input vectors so that they are uncorrelated with each other, it sorts the resulting orthogonal components (principal components) so that those with the largest variation come first, and, finally, it eliminates those components that contribute the least to the variation in the data set [23].

According to the literature [24], the most common definition of PCA is that for a set of observed vectors {*v*_*i*_},  *i* ∈ {1,2,…, *N*}, the *q* principal axes *w*_*j*_, *j* ∈ {1,2,…, *q*} are those orthonormal axes onto which the retained variance under projection is maximal. It can be shown that the vectors *w*_*j*_ are given by the *q* dominant eigenvectors (i.e., those with largest associated eigenvalues) of the covariance matrix

(1) $$\begin{array}{c} C=\sum_{i}\frac{\left(v_{i}-\bar{\nu }\right){\left(v_{i}-\bar{\nu }\right)}^{T}}{N} \end{array}$$

such that *Cw*_*j*_ = *λ*_*i*_ *w*_*j*_, where $\bar{\nu }$ is the simple mean value and *λ*_*i*_ is a scalar, termed the eigenvalue corresponding to *w*_*j*_. 

The vector $u_{i}=W^{T}(\nu_{i}-\bar{\nu })$, where *W* = (*w*_1_, *w*_2_,…, *w*_*q*_), is a *q*-dimensional reduced representation of the observed vector *ν*_*i*_ [25].

### 2.3. Least Square Support Vector Machine Classifier

Support Vector Machine (SVM) is a classification and regression prediction tool that uses machine learning theory to maximize predictive accuracy while automatically avoiding over-fit to the data. The foundations of SVMs have been developed by Vapnik [26] and gained popularity due to many promising features. SVMs perform classification by constructing an *N*-dimensional hyperplane that optimally separates the data into two categories. The goal of SVM is to produce a model in the form of *f*(*x*) = *ω* ^*T*^ *x* + *b* which predicts the target values of the test data given only the test data attributes. The training set {*x*_*i*_,*y*_*i*_}_*i*=1_^*l*^, where *x*_*i*_ ∈ *ℜ* ^*n*^ is the input and *y*_*i*_ ∈ {−1, +1} is the output, shows the class. 

The Representer Theorem [27] states that the solution *ω* can always be written as a linear combination of the training data:

(2) $$\begin{array}{c} \omega =\sum_{j=1}^{N}a_{j}y_{j}x_{j}. \end{array}$$

In that way, the SVM can be formulated to learn a linear classifier

(3) $$\begin{array}{c} f\left(x\right)=\sum_{i=1}^{N}a_{i}y_{i}K\left(x,x_{i}\right)+b, \end{array}$$

by solving an optimization problem over *a*_*i*_, where *a*_*i*_ are Lagrange, *b* is a real constant, and *N* is the size of the training data.

*K*(*x*_*i*_, *x*_*j*_) is a nonlinear kernel function given by *K*(*x*_*i*_, *x*_*j*_) = *φ*(*x*_*i*_)^*T*^ *φ*(*x*_*j*_), where *φ*(*x*) is the nonlinear map from original space to the high dimensional space.

The SVM classifiers solve the following quadratic programming problem:

(4) $$\begin{array}{c} \min\frac{1}{2}\omega^{T}\omega +C\sum_{i=1}^{N}\xi_{i} \end{array}$$

subject to *y*_*i*_(*ω* ^*T*^ *ϕ*(*x*_*i*_) + *b*) = 1 − *ξ*_*i*_, *ξ*_*i*_ ≥ 0, *i* = 1,…, *N*, *ξ*_*i*_ represents the degree of misclassification of the data *x*_*i*_ and *C* is the penalty parameter of the error term [28].

In this paper the least squares version of SVM is used, whose main advantage is that it is computationally more efficient than the standard SVM method. In this case the training process requires the solution of a linear equation set instead of the quadratic programming problem involved by the standard SVM. The LSSVM method—when Radial Basis Function (RBF) kernels are used—requires only two parameters (*C* and *σ*), while the time consumed by the training method is reduced, by replacing the quadratic optimization problem with a simple linear equation set [29]. In LSSVMs, an equality constraint-based formulation is made within the context of ridge regression as follows:

(5) $$\begin{array}{c} \min\frac{1}{2}\omega^{T}\omega +C\sum_{i=1}^{N}{e^{2}}_{i} \end{array}$$

subject to *y*_*i*_(*ω* ^*T*^ *ϕ*(*x*_*i*_) + *b*) = 1 − *e*_*i*_, *i* = 1,…, *N*.

### 2.4. The Intelligent PCA-LSSVM Prediction System

**Figure 1:**
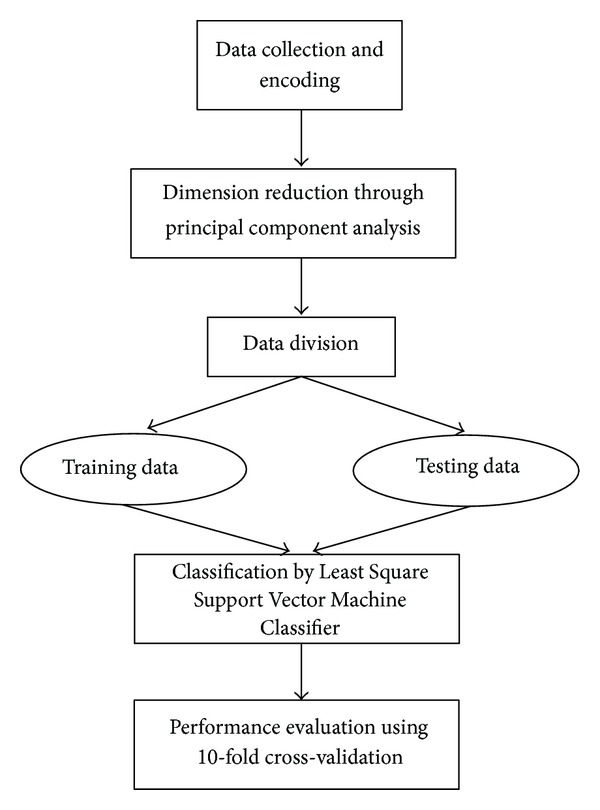
Flowchart diagram of the proposed intelligent system for asthma prediction.

The asthma prediction system which is presented in this study consists of three stages: (i) the feature extraction and dimension reduction through PCA, (ii) the pattern classification by employing LSSVM classifier, and (iii) the performance evaluation by using classification accuracy, sensitivity, specificity, and 10-fold cross-validation. The flowchart of the intelligent system for asthma prediction is illustrated in Figure 1. The implementation steps of the algorithm follow a certain sequence. First of all, the patient's data were collected and prepared in an electronic form suitable for further processing. After this step, all the parameters (where it is necessary) were encoded and the outputs were assigned either with label 1 (asthma persistence) or 0 (no asthma persistence). At last, the dimension of the dataset which had 46 features was reduced to 18 features using the PCA method.

In the classification stage of PCA-LSSVM intelligent prediction system, the reduced features obtained from the first stage were fed to the LSSVM classifier. LSSVM classifiers parameters, which are *σ* (the width of RBF kernel) and margin-losses trade-off *C*, affect the prediction performance. The best combination of *C* and *σ* was selected by the grid search with growing sequences of *C* (1–1000 with a step equals 10) and *σ* (1–100 with a step equals 1). Each combination of parameter choices was checked using 10-fold cross-validation. At first, the 112 patients were divided into 10 almost equal subgroups. One of the 10 subgroups has been used as the evaluation data and the rest as the learning data for the classification. The evaluation data were changed 10 times, so that each group was investigated once as evaluation data. The average value of all obtained accuracies of the evaluation data was considered as the estimation ability of the model. The parameters with best cross-validation accuracy were picked.

## 3. Results

The experimental results are presented in terms of accuracy, sensitivity, and specificity as shown in Table 3. The prediction is considered to be true positive (TP) if the patient has asthma and it is correctly predicted as asthmatic. On the contrary, if the asthmatic patient is incorrectly predicted as nonasthmatic, the prediction is assigned as false negative (FN) [30]. False positive (FP) and true negative (TN) predictions can be determined in the same way. Looking into the training model, there are 68 positive data (presence of asthma according to the physicians) and 44 negative (absence of asthma according to the physicians). The sensitivity, specificity, and accuracy have been estimated using the following equations:

(6) $$\begin{array}{c} \text{Sensitivity}=\frac{N_{\text{TP}}}{N_{\text{TP}}+N_{\text{FN}}}\times 100,\\ \text{Specificity}=\frac{N_{\text{TN}}}{N_{\text{TN}}+N_{\text{FP}}}\times 100,\\ \text{Accuracy}=\frac{N_{\text{TP}}+N_{\text{TN}}}{N_{\text{TP}}+N_{\text{TN}}+N_{\text{FP}}+N_{\text{FN}}}\times 100, \end{array}$$

where *N*_TP_, *N*_TN_, *N*_FP_, *N*_FN_ are the number of TP, TN, FP, FN, respectively [31]. Sensitivity and specificity are statistical measures of the performance of a binary Classification test. Sensitivity measures the percentage of positive (asthmatic) people that have been correctly identified as having asthma. Specificity measures the percentage of negative (not asthmatic) people which have been correctly identified as not having asthma. The accuracy is the degree of how close the predicted values are to the actual ones.

In Table 3, the best-performed 10 combinations of *C* and *σ* values and the correct asthma prediction rates are presented. As it can be seen from these results, the value having the highest prediction accuracy for the proposed asthma prediction intelligent method was found to be 95.54%, for the case where *σ* = 7 and *C* = 10. 

## 4. Discussion

The predictive accuracy of the proposed system is not easily comparable with that of other studies because of differences in study design and objectives. To the authors' knowledge a limited number of studies have been published on asthma prediction in children at the age when the symptoms are observed. In the study of Caudri et al. [32] the asthma prediction was based on eight clinical parameters, considering children from 7 to 8 years of age. These eight parameters were male sex, postterm delivery, parental education and inhaled medication, wheezing frequency, wheeze/dyspnea apart from colds, respiratory infections, and eczema. In 72% of the cases, the model accurately discriminated the asthmatic and the nonasthmatic children. Clough et al. [33] have developed models to examine the potential risk factors for wheeze that persists for at least 12 months after presentation in a group of young children, each with at least one atopic parent, with early-life wheezing. This paper has shown that increased age at presentation, personal atopy and raised soluble IL-2R are all associated with increased risk. Castro-Rodríguez et al. [34] developed two clinical indices at 3 years of age for the prediction of asthma in school age. It was shown that 59% of children with a positive loose index and 76% of those with a positive stringent index had active asthma in at least one survey during the school years. Their indices include characteristics of wheezing during the first 3 years of life, parental asthma or eczema, wheezing without colds, eosinophilia, or allergic rhinitis. Finally, in the study of Devulapalli et al. [35] the number of hospital admissions for obstructive airways disease within the first 2 years of life has been included in the predictive model, giving positive predictive values and negative predictive value of 55% and 92%, respectively.

Based on the comparison, which has been already shown above, it seems that there are valuable studies published on asthma prediction. However, these prediction methods are not able to achieve substantially high predictive accuracies. It is, therefore, meaningful to utilize computational intelligence methods in order to overcome such problems. Such an example has been shown in this paper. The proposed method for asthma prediction up to the age of 5 might predict the asthma with an accuracy exceeding 95%. However future studies should be performed to further evaluate our proposed method in clinical practice. Moreover, regardless of the prediction outcome using the presented algorithm, an evaluation of the results in cooperation with medical doctors who are asthma specialists must be performed in order to decide if either the patient needs treatment or not.

## 5. Conclusions

In this paper, a new intelligent system based on the Principal Component Analysis and Least Square Support Vector Machine classifier for asthma prediction has been proposed. The used parameter vector had a significantly high dimensionality and it was, therefore, necessary to be reduced in order to achieve as low computational cost as possible on the one hand, while on the other hand to minimize the complexity of the system. Due to the fact that asthma is a serious health condition, the various models, which have been used to detect it, must have high accuracy so that patients with asthma are not overlooked. 

The experimental results show that the proposed method can predict 95.54% of patients with asthma. To conclude, the proposed system can give a significant contribution and be a useful tool in clinical practice for the physicians in order to overcome many of the therapeutic dilemmas. 

## Figures and Tables

**Table 1:** Prognostic factors.

Category	Prognostic factors
Demographic	age, sex, ethnicity^#^, height, weight, waist's perimeter, residence^#^
Wheezing episodes	until 3rd year, between 3rd and 5th year
Symptoms	wheezing*, cough*, allergic rhinitis*, runny nose*, congestion*, eczema*, food allergy*, pharmaceutical allergy*, allergic conjunctivitis*, dyspnea*, seasonal symptoms^#^
Parental history	asthma*
House conditions	number of family members, pets*, type of heating^#^
Pharmaceutical therapy	bronchodilators, corticosteroids inhaled*, corticosteroids per os*, antileukotriene*, antihistamine*
Breathing tests	FEV_1_%, FEF_25/75_%
Tests	Ig E U/Ml
Allergens	*d*. *pteronyssinus* ^#^, *d*. *farinae* ^#^, olive^#^, pellitory^#^, graminaceae^#^, pine^#^, cypress^#^, cat^#^, dog^#^, *alternaria* ^#^
Neonatal period	pregnancy duration, breastfeeding duration^#^, smoking during pregnancy*
Asthma	treatment*

*The encoding is binary: yes (1) or no (0).

^#^The encoding is shown in Table 2.

All other factors are numerical.

**Table 2:** Encoding of some prognostic factors.

Prognostic factor	Coding					
Sex	0 (Male)	1 (Female)				
Residence	0 (Urban)	1 (Semiurban)	2 (Rural)			
Season of the symptoms	0 (None)	1 (Winter)	2 (Autumn)	3 (Spring)	4 (Summer)	5 (>2 Seasons)
Type of heating	0 (Central heating)	1 (Wood stove)	2 (Oil stove)	3 (Fireplace)	4 (Central heating + Fireplace)	
Pregnancy duration in weeks	0 (<37)	1 (37-38)	2 (>38)			
Allergens	0 (0)	1 (3.5–6 mm)	2 (>6 mm)			

**Table 3:** Accuracy, sensitivity, and specificity percent values for 10 combinations of *C* and *σ*.

Selection of *σ* parameter	Selection of *C* parameter	Accuracy	Sensitivity	Specificity
30	10	93.75	97.73	91.18
28	100	94.64	97.73	92.65
62	100	93.75	97.73	91.18
**7**	**10**	**95.54**	**95.45**	**95.59**
15	10	94.64	97.73	92.65
72	1000	93.75	97.73	91.18
49	100	93.75	97.73	91.18
58	10	90.18	95.45	86.76
25	10	93.75	97.73	91.18
50	1000	94.64	97.73	92.65
